# Posterior scleral deformation and autonomic dysfunction in normal tension glaucoma

**DOI:** 10.1038/s41598-020-65037-6

**Published:** 2020-05-18

**Authors:** Da Young Shin, Soo Ji Jeon, Hae Young Lopilly Park, Chan Kee Park

**Affiliations:** Department of Ophthalmology, Seoul St. Mary’s Hospital, College of Medicine, The Catholic University of Korea, 222 Banpo-daero, Seocho-gu, Seoul, 06591 Republic of Korea

**Keywords:** Optic nerve diseases, Risk factors

## Abstract

In meta-analyses, it has been reported that myopia is a risk factor for glaucoma and there is increasing evidence that autonomic dysfunction causing vascular dysregulation or perfusion dysfunction is considered an important factor in the progression of glaucoma. There have been experimental studies to find out the association between autonomic nervous system and ocular growth, but no clinical study yet has evaluated the relationship between them. Therefore, we enrolled 208 open angle glaucoma patients and measured heart-rate-variability(HRV). We used the standard deviation value of the qualified normal to normal intervals (SDNN) parameter of HRV, which is considered an autonomic influence index and characterized the total effect of the regulation of autonomic blood circulation. Patients were classified into the two groups according to SDNN: those with low possibility of autonomic dysfunction (LoAD group) and those with high possibility of autonomic dysfunction (HiAD group). We evaluated myopic features employing a ‘posterior scleral profile’ identified by the disc tilt ratio, disc torsion, fovea-BMO center (FoBMO) angle and peripapapillary area(PPA) to disc ratio. HiAD group showed higher values than LoAD group in posterior scleral deformation profile such like axial length, disc tilt, torsion degree. We suggest the possibility of association between myopic deformation and autonomic dysfunction.

## Introduction

Glaucoma is a progressive optic neuropathy characterized by the optic nerve damage and loss of retinal ganglion cells^1^. While elevated intraocular pressure and age are considered main risk factors for the development and progression of the disease, the pathogenesis of glaucoma is still unclear. There is increasing evidence that vascular dysfunction and perfusion insufficiency need to be considered as important factors in the progression of glaucoma^2–4^. Autonomic dysfunction may contribute to unstable or fluctuating blood pressure which can cause dysfunction of autoregulation and lead to glaucoma progression^5^. A previous study revealed that there is the sympathovagal inbalance of autonomic nervous system(ANS) in patients with NTG^6–8^. Sympathovagal inbalance may be responsible for the faster rate of central visual field(VF) progression in NTG^9^.

Meanwhile, myopia has been reported as a risk factor for glaucoma in meta-analyses of epidemiological studies^10^. The Tajimi Study reported that age, intraocular pressure (IOP) and myopia were significant risk factors for primary open-angle glaucoma (POAG) and normal-tension glaucoma (NTG)^11^. In parallel with the increased prevalence of myopia, glaucoma also increases. Posterior pole profile variables like disc tilt, FoBMO angle, disc torsion, PPA to disc ratio are related to myopic optic nerve change^12,13^. These structural changes in the ONH may affect the higher susceptibility to glaucomatous optic disc damage^14,15^. Brazitikos *et al*.^16^ reported that tilted disc may cause visual field defects. Park *et al*.^17^ reported that optic disc torsion was related to the location of the visual field defect.

Several experimental studies have documented that there is relationship between the autonomic nervous system and ocular growth^18–20^. They reported that dysfunction or abnormality of autonomic nervous system result in ultradian rhythms in axial length and choroidal thickness that are associated with an increased rate of ocular growth underlying myopia development^18^. But no study yet has evaluated the linkage of myopic ocular change and autonomic inbalance in glaucoma patients, which are known to be associated with glaucomatous optic nerve damage.

The purpose of the present investigation was to evaluate whether the various indices including the posterior scleral profile have differences in according to presence of autonomic dysfunction.

## Results

The mean value of the SDNN in 208 NTG patients was 33.71 and the standard deviation was 18.16. So LoAD group had a SDNN values between 15.55(33.71 –(minus) 18.16) and 51.87(33.71 + (plus)18.16) and HiAD group had a SDNN value less than 15.55 or greater than 51.87. Among 208 NTG patients, 164(78.8%) were LoAD group (15.55–51.87) and 44(21.2%) were HiAD group (<15.55 or >51.87). Analysis of several factors, including the posterior scleral pole profile for both groups is shown in Table 1. There were no significant differences between LoAD group and HiAD group with respect to age, sex, CCT, spherical equivalent, MD of VF, PSD of VF, average RNFL thickness. Axial length was significantly longer in HiAD group than LoAD group (p = 0.022). Torsion degree was significantly higher in HiAD group than LoAD group (p < 0.001), and the only factor related to heart rate variability in both simple (p < 0.001) and multiple (p = 0.008, p = 0.040 after bonferroni correction) logistic regression analysis (Table 2).

**Table 1:** Comparison of the Demographics and test result between LoAD and HiAD group.

Variables	LoAD group, 164 eyes 164 patients	HiAD group, 44 eyes 44 patients	P value
Age, y	53.29 $\pm$12.51	52.45 $\pm$13.89	0.700*
Sex, male/female	58/106	14/30	0.660^†^
CCT, μm	528.06 $\pm 33.64$	519.41 $\pm 47.58$	0.322*
history of refractive surgery, n(%)	10 (6.1%)	4 (9.1%)	0.482^†^
history of diabetes, n(%)	6 (3.7%)	0 (0.0%)	0.198^†^
history of hypertension, n(%)	16 (9.8%)	6 (13.6%)	0.457^†^
history of CVA, n(%)	4 (2.4%)	2 (4.5%)	0.459^†^
Migrain, n(%)	34 (20.7%)	11 (25.0%)	0.541^†^
Spherical equivalent, D	−3.15 $\pm 3.83$	−3.78 $\pm 5.21$	0.461*
Axial length, mm	25.04 $\pm 1.60$	25.94 $\pm 2.23$	0.022*
PPA/Disc area, per 1 µm larger (%)	54.04 $\pm 84.70$	55.32 $\pm 54.84$	0.926^†^
Tilt ratio	1.21 $\pm 0.18$	1.28 $\pm 0.22$	0.049^†^
Torsion degree	5.99 $\pm 6.75$	11.60 $\pm 11.52$	0.005^†^
FoBMO angle	−6.33 $\pm 3.56$	−5.83 $\pm 3.99$	0.420*
Disc hemorrhage, n (%)	28 (17.1%)	6 (13.6%)	0.584^†^
VF MD, dB	−4.54 $\pm$4.69	−5.63 $\pm$5.86	0.199*
VF PSD, dB	5.40 $\pm$4.34	4.93 $\pm$4.29	0.796*
Average RNFL thickness	77.97 $\pm$12.25	74.34 $\pm$11.26	0.078*

Data are presented as mean $\pm$ standard deviation.

CCT = central corneal thickness; CVA = cerebrovascular accident; D = diopter; PPA = peripapillary atrophy; FoBMO = fovea-BMO center; VF = visual field; MD = mean deviation; PSD = pattern standard deviation; RNFL = retinal nerve fiber layer.

Statistically significant values (p < 0.05) appear in boldface.

*Comparison was performed using independent samples t-test.

^†^Comparison was performed using a chi-squared test and Fisher’s exact test.

**Table 2:** Logistic regression results for factors associated with the autonomic dysfunction (n = 208).

Variables	Simple model		Multiple model	
	Odds Ratio, 95% CI	P value	Odds Ratio, 95% CI	P value
Age, y	0.99, 0.97–1.02	0.699		
Sex, male/female	1.17, 0.58–2.39	0.661		
CCT, μm	0.99, 0.98–1.00	0.218		
history of refractive surgery, n(%)	1.54, 0.46–5.17	0.485		
history of diabetics, n(%)	0.00, 0.00–0.00	0.999		
history of hypertension, n(%)	1.46, 0.54–3.99	0.459		
Self reported history of CVA, n(%)	1.91, 0.34–10.76	0.466		
Migrain,n(%)	1.28, 0.58–2.78	0.542		
Spherical equivalent, D	0.97, 0.90–1.04	0.379		
Axial length, mm	1.32, 1.08–1.63	0.008*	0.85, 0.67–1.09	0.203
PPA/Disc area, per 1 µm larger (%)	1.02, 0.67–1.55	0.926		
Tilt ratio	4.64, 0.95–22.72	0.058*	0.33, 0.05–2.20	0.251
Torsion degree	1.07, 1.03–1.11	<0.001*	0.94, 0.90–0.98	0.008**
FoBMO angle	1.04, 0.95–1.14	0.418		
Disc hemorrhage, n (%)	0.77, 0.30–1.99	0.585		
VF MD, dB	0.96, 0.90–1.02	0.200*	0.95, 0.86–1.05	0.329
VF PSD, dB	0.99, 0.92–1.07	0.836		
Average RNFL thickness	0.98, 0.95–1.00	0.080*	1.01, 0.97–1.05	0.608

CCT = central corneal thickness; CVA = cerebrovascular accident; D = diopter; PPA = peripapillary atrophy; FoBMO = fovea-BMO center; VF = visual field; MD = mean deviation; PSD = pattern standard deviation; RNFL = retinal nerve fiber layer.

Statistically significant values appear in boldface.

*p < 0.2.

**Statistically significant values, p = 0.04 after Bonferroni correction.

## Discussion

To the best of our knowledge, this is the first study that has revealed a relation with myopic ocular change and autonomic nervous system in patients with glaucoma. The association of these two factors with glaucoma has already been well investigated, respectively.

An association between open-angle glaucoma and myopia has been reported for decades^21^. There have been many studies to explain the correlation between glaucoma and myopia^22^. In myopia, structures of optic nerve, including lamina, may be more vulnerable to glaucomatous damage. The shearing force caused by scleral deformation of myopia affects the lamina cribrosa^23–27^. This may be the main mechanism of glaucomatous damage in myopia^23–27^. There are also studies about myopia-related optic disc and retinal change such like the axis of tilt and tilt ratio and PPA^28^. Chameen *et al*.^29^ reported that beta-PPA was predominantly located in the temporal direction in eyes with disc tilt as myopic patients are predominantly temporally tilted. Tilted disc may exacerbate visual field defects^16^. Park *et al*.^17^ reported that the torsion direction of the optic disc may predict the location of VF damage. So, the indicators of myopic optic disc change are associated with glaucomatous damage.

There are many studies that hemodynamic instability can contribute to the onset and exacerbation of glaucoma^30–32^. Glaucoma patients with vascular dysregulation have impaired autoregulatory function, so they cannot respond appropriately to low perfusion pressure^33,34^. The pathogenesis of vascular dysregulation is not clearly known, but it is assumed to be due to dysfunction of ANS and endothelial vascular layer^35^. Systemic sympathetic or parasympathetic neuropathy has been reported in patients with POAG and NTG patients^36–38^. In particular, NTG patients have been reported to have abnormal autonomic nervous system, it would have affected ocular blood flow and structural damage^8^.

In our study, we described the correlation between myopic change parameter, namely the posterior scleral profile, and an autonomic dysfunction parameter, namely HRV. Nickla *et al*.^18,39^ tried to explain the relationship between myopia and autonomic nervous system by several experiments in chicks. They found abnormal ocular rhythms in chick having procedures like parasympathetic resection, which causes autonomic dysfunction^18^. They also reported that abnormal ocular rhythm results in abnormal ocular growth stimulation^18^.

We used Heart rate variability (HRV) to evaluate autonomic dysfunction. HRV is a non-invasive tool, which is widely used to evaluate the autonomic nervous system (ANS). HRV provides information about the functions of the sympathetic and parasympathetic nervous system^40^. HRV is defined as ‘the amount of fluctuations from the mean heart rate’^41^. It is mainly controlled by the continuous interaction of the ANS^41^. The HRV test has many indicators. Among them, the SDNN is considered the best indicator of the ANS^42^. It reflects all the cyclic components that affect variability, therefore it represents total variability and balance of SNS and PNS^43^. Autonomic nervous system dysfunction which can cause blood pressure problems may relate to hypertension, orthostatic intolerance, cardiac disease, and other disease states^44^. So it can cause fluctuation of blood pressure. When blood pressure is decreased and renal blood flow is decreased, juxtaglomerular cells convert the precursor prorenin into renin in the kidneys^45^. Renin breaks down angiotensinogen synthesized in the liver to make angiotensin I^46^. Angiotension I is converted to angiotensin II^46^. This angiotensin II has multiple effects in various organs including constricting blood vessels and producing aldosterone in the adrenal cortex^45,46^.

Meanwhile, it has been reported that ECM synthesis is regulated by sympathetic nervous system or angiotensin II^47^. So activated renin-angiotension system(RAS) affects the ECMs in many organs, then what about eyeballs? Sclera is composed of approximately 50% collagens and ECM such as proteoglygan, glycoproteins and matrix metalloproteinase. Therefore, We hypothesize that the renin angiotension system may have a effect on sclera. Especially events that occur in scleral extracellular matrix during childhood and young adulthood may be very important because it is the time when the eyeball is growing. The composition of extracellular matrix plays a very important role in determining the properties of the sclera, which affects the eyeball elongation^48^. Deformity of scleral canal (disc tilt, torsion, PPA) and thinning of sclera, especially posterior pole, is usually happens with elongation of the eyeball during childhood and young adulthood. We carefully suggest that people who have abnormality of HRV are more affected by activated renin-angiotensin system than normal people during childhood and young adulthood. So that may explain our result that posterior scleral deformity such as tilt ratio, FoBMO angle, axial length is associated with abnormality of HRV.

In our results, there was no statistical difference in refraction (spherical equivalent) between the two groups. The amount of refraction is determined by axial length, curvature and refractive index. Curvature myopia refers to a type of refractive error that develops when the cornea is curved too much. By contrast, index myopia is caused by change in the refractive index of the media such as cataracts or hydration of lens (diabetes, drugs)^49^. The pathophysiology of myopia that we are interested in is not just myopia (myopic refraction), but axial myopia. Because only axial myopia is associated with posterior scleral deformity and glaucomatous optic nerve damage.

We found a difference for axial length, tilt ratio and torsion degree of the optic disc in Chi-Square, independent t-test and logistical regression analysis, with only torsion degree being statistically significant for a multivariate(multiple) logistic regression analysis. It does not mean that the axial length, tilt ratio are not associated with HRV. Since all three factors: axial length, tilt and torsion, have similar mechanism in development, the association wound have been reduced in multivariate analysis, which adjust for confounding.

**Figure 1:**
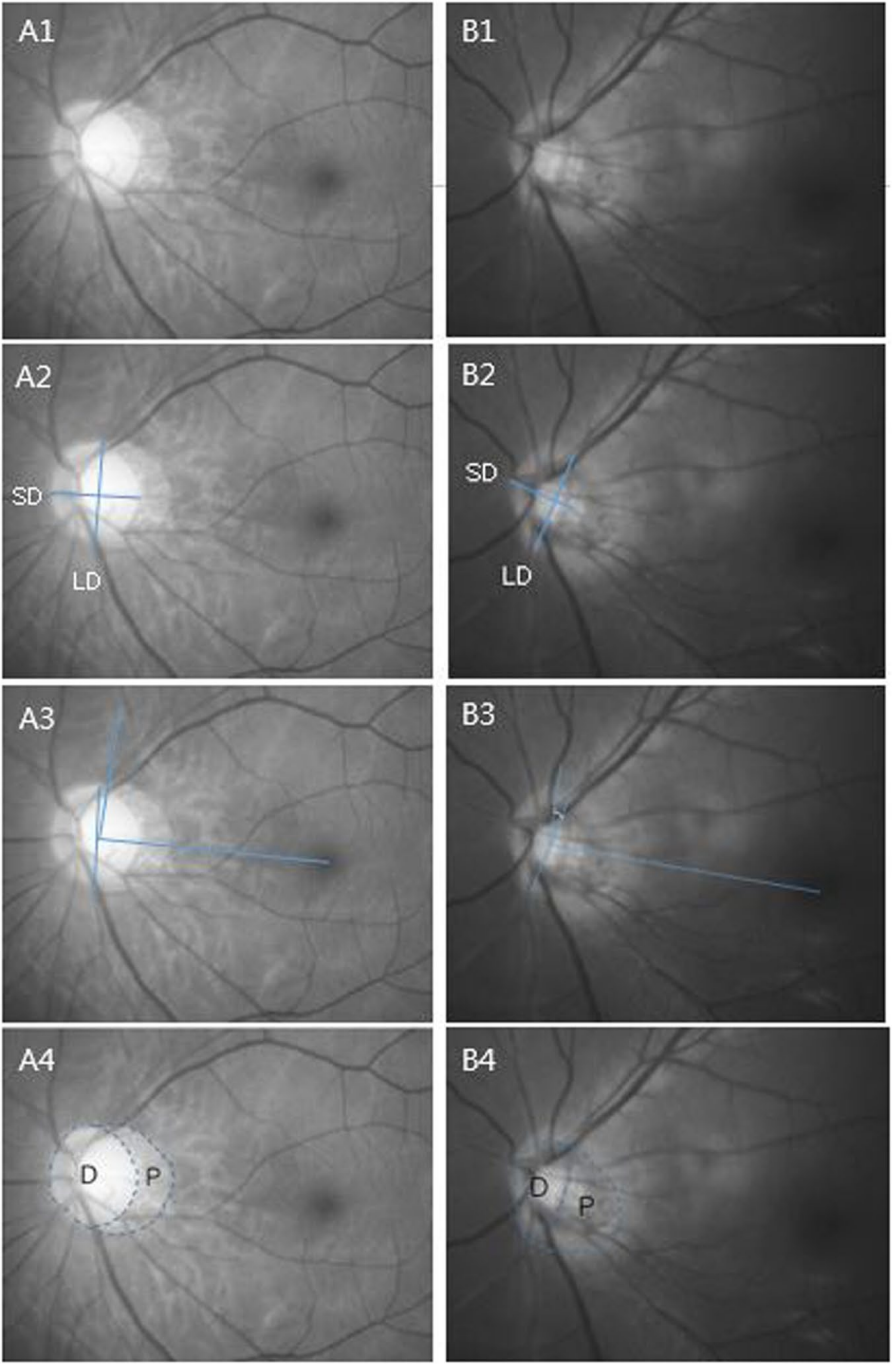
A representative case. A1. A 46-year-old woman. Her SDNN of HRV was 34.87. She was classified as LoAD group. Her axial length was 28.31 mm. B1. A 32-year-old man. His SDNN of HRV was 14.71. He was classified as HiAD group. His axial length was 27.93 mm. A2. Tilt ratio was defined as the ratio between the longest diameter (LD) and the shortest diameter (SD) of the optic disc. Tilt ratio of LoAD group patient was 1.10. B2. Tilt ratio of HiAD group patient was 1.92. A3. Torsion degree was measured between the LD and the horizontal line connecting the fovea and the center of the optic disc. Torsion degree of LoAD group patients was 6.5 $^\circ$. B3. Torsion degree of HiAD group patients was 17.5 $^\circ$. A4. PPA/disc ratio was defined as the ratio between area of D and area of P. PPA/disc ratio of LoAD group patients was 0.51. B4. PPA/disc ratio of HiAD group patients was 0.96.

Figure 1 shows representative case. The patient of HiAD group shows a higher tilt ratio, torsion degree and PPA to disc ratio than patient of LoAD group.

In summary, We found that patients with abnormality of HRV had more association with posterior scleral deformity such as disc tilt, FoBMO angle, axial length. And further studies are needed to explore this finding and mechanism.

## Material and Methods

### Patients

This study included 208 OAG patients who visited the Seoul St. Mary’s Hospital between September 2018 and October 2018. It was approved by Institutional review and Ethics Board of Seoul St. Mary’s Hospital and performed according to the tenets of the Declaration of Helsinki. Written informed consent was obtained from all the subjects.

All patients enrolled underwent comprehensive ophthalmologic examination consisting of best-corrected visual acuity, intraocular pressure by Goldmann applanation tonometry. Anterior chamber angle evaluated by gonioscopy. Central corneal thickness were measured using ultrasound pachymetry (Tomey Corp, Nagoya, Japan), and axial length was measured by biometry (IOLMaster, Carl Zeiss Meditec, Dublin, CA, USA). Optic disc was examined with red-free fundus photography (Kowa nonmyd WX; Kowa Company Ltd., Tokyo,Japan). RNFL thickness was measured using the Cirrus OCT(Cirrus, version 6.0; Carl Zeiss Meditec). Global RNFL thickness was automatically calculated. VF was examined using the Swedish Interactive Threshold Algorithm standard 24-2 perimetry (Carl Zeiss Meditec, Dublin, CA, USA). History of disc hemorrhage (DH) was investigated through medical records. NTG was defined as the presence of an open anterior chamber angle, glaucomatous optic disc cupping by funduscopic examination and compatible repeatable VF damage, and IOP never exceeded 21 mmHg by a Goldmann applanation tonometer in repeated measurement on different days. Glaucomatous optic disc is increased cupping or loss(focal/diffuse) of neuroretinal rim. Glaucomatous VF was defined as a cluster of 3 or more non-edge points on pattern deviation map with a probability <5% of the healthy population, including at least one of these points with the probability of <1% of the healthy population; or VF is outside the normal limits on the glaucoma hemifield test for a pattern standard deviation (PSD) outside 95% normal limits, reliable test (<15% false negatives, <15% false positives and ≤33% fixation losses). Refractive errors (myopia, hyperopia, presbyopia) were usually corrected with a trial lens when performing VF tests. Mild myopia not exceeding −3 diopters was not corrected, astigmatism less than 1 diopter was corrected with spherical equivalent and astigmatism over −1.25 diopters was corrected with the corrective lens. And in myopia over −6 diopters, contact lenses were used when VF examination was performed^50^. Exclusion criteria included any other neurologic disorder and the taking of any drugs known to affect ANS or the cardiovascular system.

### HRV assessment

The participants were asked to avoid activities that could affect blood pressure at least 2 hours before the test. The testing was performed over 5 minutes in controlled conditions. The echocardiography was monitored for 5 minutes by an experienced technician. The echocardiography signal was transferred to a Medicore Heart rate Analyzer, Model SA-3000P (Medicore, Seoul, Korea). The SDNN index is the mean of the standard of all the normal RR ECG wav intervals of the standard deviations of all the normal RR intervals. The parameter is primarily a measure of the autonomic effect on heart rate variability and characterize the total effect of autonomic blood circulation regulation^43^. A reduction of SDNN is correlated with left ventricular dysfunction indicating a high tone of heart sympathetic activity and has been found to be predictive of increased risk of sudden cardiac death^51–55^. Reduced SDNN mainly reflects sympathetic overactivity, and increased SDNN reflect parasympathetic overactivity^56^.

Patients were divided into 2 groups, according to their SDNN. LoAD group consisted of patients within the one standard deviation from the average of the SDNN measurements. And HiAD group consisted of patients outside one standard deviation from the average of the SDNN measurements, indicating that they had too higher or too lower SDNN of HRV. So HiAD group having deviated value was thought to have abnormality in HRV. it can cause fluctuation of blood pressure.

### Posterior scleral profile (optic disc tilt, torsion, PPA to disc ratio, FoBMO angle)

Optic disc tilt, torsion, and PPA to disc ratio were measured from photographs by a two independent examiners(DYS and H-YLP) using the National Institutes of Health image-analysis software (ImageJ version 1.40; available at http://rsb.info.nih.gov/ij/index.html; developed by Wayne Rasband, National Institutes of Health, Bethesda, MD).

Optic disc tilt was defined as the ratio between the longest and shortest diameters of the optic disc (Figure 1A2,B2)^13,17,57,58^. The torsion of optic disc was identified and defined as the deviation of the long axis of the optic disc from the vertical meridian (Figure 1A3,B3)^12,17,28^. The technique of assessing the disc tilt and torsion has already been described and applied in previous investigations^12,13,17,28,57,58^.

The pixel areas of the PPA were calculated using the imageJ software, and the pixel area of the disc were also calculated. And PPA to disc ratio defined as the ratio between the PPA area and disc area(PPA/disc ratio = PPA area/disc area)(Figure 1A4,B4).

FoBMO angle determined as the angle between and the line connecting the fovea and BMO center and the horizontal meridian passing through the Bruch’s membrane opening (BMO) center. It was measured by spectral domain-OCT scans performed with the Heidelberg spectralis OCT(spectralis software v. 5.1.1.0, Eye Explorer Software 1.6.1.0; Heidelberg Engineering).

### Statistical analysis

Sample size calculations were performed using the G*Power 3.1. The sample size calculation showed a power of 80 and alpha error of 0.05, for sample size of 160 in the LoAD group versus 40 in the HiAD group. The independent t-test and chi-square test for independent samples were used to assess the differences between groups. Binary logistic regression analyses were used to identify posterior scleral factors associated with autonomic dysfunction. A value of P < 0.2 in the simple binary logistic regression model was included in multiple model. A value of P < 0.05 indicated statistical significance. The independent variables were age, axial length, MD of the VF, PSD of the VF, PPA to disc ratio, FoBMO angle from spectral domain-OCT, tilt ratio, torsion degree. All statistical analysis were performed with SPSS for windows statistical software(ver.24.0; SPSS Inc, Chicago, Illinois, USA).
